# Platform combining statistical modeling and patient-derived organoids to facilitate personalized treatment of colorectal carcinoma

**DOI:** 10.1186/s13046-023-02650-z

**Published:** 2023-04-03

**Authors:** George M. Ramzy, Maxim Norkin, Thibaud Koessler, Lionel Voirol, Mathieu Tihy, Dina Hany, Thomas McKee, Frédéric Ris, Nicolas Buchs, Mylène Docquier, Christian Toso, Laura Rubbia-Brandt, Gaetan Bakalli, Stéphane Guerrier, Joerg Huelsken, Patrycja Nowak-Sliwinska

**Affiliations:** 1 Molecular Pharmacology Group, School of Pharmaceutical Sciences, University of Geneva, Rue Michel-Servet 1, CMU, 1211 Geneva 4, Switzerland; 2 Institute of Pharmaceutical Sciences of Western Switzerland, University of Geneva, 1211 Geneva, Switzerland; 3 Translational Research Center in Oncohaematology, 1211 Geneva, Switzerland; 4 Swiss Institute for Experimental Cancer Research (ISREC), Ecole Polytechnique Fédérale de Lausanne-(EPFL-SV), 1015 Lausanne, Switzerland; 5 Department of Oncology, Geneva University Hospitals, 1205 Geneva, Switzerland; 6 Research Center for Statistics, Geneva School of Economics and Management, University of Geneva, 1205 Geneva, Switzerland; 7 Division of Clinical Pathology, Diagnostic Department, University Hospitals of Geneva (HUG), 1205 Geneva, Switzerland; 8 Translational Department of Digestive and Transplant Surgery, Geneva University Hospitals and Faculty of Medicine, 1205 Geneva, Switzerland; 9 iGE3 Genomics Platform, University of Geneva, 1211 Geneva, Switzerland; 10 Department of Genetics & Evolution, University of Geneva, 1211 Geneva, Switzerland; 11 Department of Visceral Surgery, Geneva University Hospital, 1211 Geneva, Switzerland; 12 EMLYON Business School, Artificial Intelligence in Management Institute, Ecully, France

**Keywords:** Drug-drug interaction, Drug resistance, Multidrug combination, Organoid, Phenotypic screen, Synergy, Targeted RNAseq

## Abstract

**Background:**

We propose a new approach for designing personalized treatment for colorectal cancer (CRC) patients, by combining ex vivo organoid efficacy testing with mathematical modeling of the results.

**Methods:**

The validated phenotypic approach called Therapeutically Guided Multidrug Optimization (TGMO) was used to identify four low-dose synergistic optimized drug combinations (ODC) in 3D human CRC models of cells that are either sensitive or resistant to first-line CRC chemotherapy (FOLFOXIRI). Our findings were obtained using second order linear regression and adaptive lasso.

**Results:**

The activity of all ODCs was validated on patient-derived organoids (PDO) from cases with either primary or metastatic CRC. The CRC material was molecularly characterized using whole-exome sequencing and RNAseq. In PDO from patients with liver metastases (stage IV) identified as CMS4/CRIS-A, our ODCs consisting of regorafenib [1 mM], vemurafenib [11 mM], palbociclib [1 mM] and lapatinib [0.5 mM] inhibited cell viability up to 88%, which significantly outperforms FOLFOXIRI administered at clinical doses. Furthermore, we identified patient-specific TGMO-based ODCs that outperform the efficacy of the current chemotherapy standard of care, FOLFOXIRI.

**Conclusions:**

Our approach allows the optimization of patient-tailored synergistic multi-drug combinations within a clinically relevant timeframe.

**Graphical Abstract:**

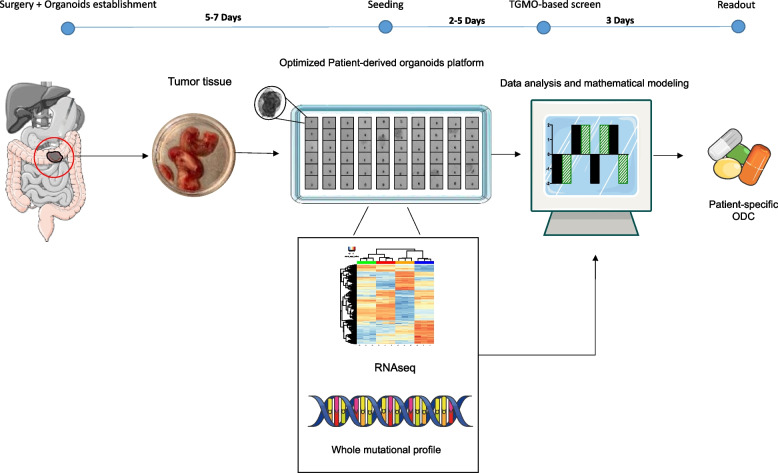

**Supplementary Information:**

The online version contains supplementary material available at 10.1186/s13046-023-02650-z.

## Introduction

Colorectal cancer (CRC) is the third most diagnosed cancer worldwide with incidence exceeding a million of newly diagnosed cases per year [1]. CRC originates from an uncontrolled cellular growth in the epithelial layer of the colon. These lesions begin with hyperplasia, and before becoming malignant they are known as polyps and can be removed during regular colonoscopy (local excision). However, if untreated, adenomatous polyps can become dysplastic and further develop into a carcinoma. When localized—stage I–III CRC surgical resection is the main treatment modality. Patients with stage II (high-risk) and stage III CRC should receive adjuvant chemotherapy (post-surgery chemotherapy) usually given for three to six months to decrease the risk of relapse.

For treatment decisions, testing for mismatch repair (*MMR*) status and mutations in *KRAS, BRAF*, and *NRAS* is recommended in all patients with metastatic disease (mCRC). Stage IV patients with mCRC are treated with a combination of chemotherapy (e.g. FOLFOX/FOLFIRI/FOLFOXIRI) plus a drug targeting VEGF (i.e., bevacizumab) or EGFR (i.e., cetuximab) [1]. The use of EGFR inhibitors remains conditional on the absence of *NRAS/KRAS/BRAF* mutations. Bevacizumab and cetuximab have been simultaneously administered with chemotherapy in mCRC, but this approach decreases the quality of life and does not improve overall median survival [2]. For the rare mCRC patients with liver-only lesions amenable to surgery, the treatment sequence usually starts with neo-adjuvant chemotherapy before surgery. Testing for microsatellite instability (MSI) is routinely used in mCRC tumors [3]. MSI is a consequence of deficient DNA mismatch repair mechanisms, leading to an increased mutational burden and higher immune cell infiltration [4, 5]. Only 15% of localized CRC and 5% of mCRC tumors are MSI and can eventually benefit from immunotherapy (i.e., pembrolizumab or nivolumab) [6]. An international consortium has created a molecular classification of CRC. Based on gene expression, tumor microenvironment (TME) and the immune landscape, four molecular subtypes of CRC have been defined as consensus molecular subtypes (CMS1-4) of CRC [7]. Furthermore, the stromal component of the tumor has been shown to strongly influence the cancer cell intrinsic transcriptional features and prognosis. Therefore, five CRC intrinsic subtypes have been recently identified (CRIS A-E) [8].

A “one-size fits all” treatment approach is employed in each of the disease stages, with limited efficacy especially for late-stage CRC, mainly due to acquired drug resistance and inadequate choice of treatment. The advent of targeted therapies considerably changed the clinical management of CRC treatment. However, their efficacy remains limited mainly due to toxicity of the relatively high doses and the targeting of mainly one signaling pathway [9]. When targeting a signaling pathway, the cells may compensate such a blockade by activating other signaling pathways [10]. This paved the way for poly pharmacology, which allowed to tackle the complex machinery of cancer by targeting multiple signaling pathways orchestrating the disease resistance [11]. Furthermore, such approach may limit toxicities significantly, as well as induction of drug resistance. This can be achieved by combining drugs targeting different signaling pathways or designing a drug combination that acts on multiple targets with various affinities [12]. The development of such therapies is still in its early age and faces a lot of challenges, as our knowledge on the drug-drug interactions in complex disease environment is far from being fully elucidated.

Challenges include the selection of the drug candidates and the decision on which cellular signaling pathways to target to enhance efficacy and limit toxicity, as well as occurrence of resistance [13]. To date, the treatment of CRC still relies heavily on conventional chemotherapy, where drug-drug interaction and selectivity is not optimal. We have previously shown that within FOLFOXIRI, a chemotherapy combination containing (folinic acid [0.5 µM], 5-fluorouracil [10 µM], SN38 (active metabolite of irinotecan) [0.1 µM] and oxaliplatin [0.5 µM]), the synergy only resides between 5-fluorouracil and folinic acid, however, the interactions between the other agents are only additive or even antagonistic [14].

In order to reliably identify an optimized and personalized treatment, the selection of an appropriate ex vivo model is of extreme importance [15, 16]. The technology is on a tight time constraint with the need to deliver results in 2–3 weeks, which corresponds clinically to the average time between a diagnosis and treatment decision. The models should be rapidly established and “ready to operate” in a clinical timeframe, which is inaccessible for animal models. Patient-derived organoids (PDOs) emerged as an effective ex vivo drug screening tool in cancer drug discovery. Over the past decade, multiple studies have highlighted the capacity of PDOs to predict clinical treatment responses in their corresponding patients [17–19]. With the recent advances in genomic sequencing and molecular biology detection techniques, we start witnessing the dawn of cancer precision medicine.

The identification of the most potent drug combination that would be both efficient and safe, is not trivial. The use of a proprietary platform, developed in our laboratory, called therapeutically guided multidrug optimization (TGMO), enables rapid identification of optimized drug combinations (ODCs) from a large set of available possibilities [20, 21]. This phenotypic approach, which uses very limited experimental testing and in silico data modelling allowed the discovery of potent and selective drug combinations [21–23] that were further successfully validated in relevant in vivo tumor models [21].

We performed a TGMO-based drug screen using CRC complex models, i.e., co-cultures of chemotherapy-naïve and -resistant cell lines, as well as freshly isolated CRC organoids. We identified ODCs specific to different cell types. Those ODCs proved to overcome the activity of FOLFOXIRI at clinically used doses. This, t with whole-exome sequencing and RNAseq data, validated applying the TGMO-based screening method directly on freshly isolated PDOs. We favor translational development of the proposed technology.

## Results

### Phenotypic screen reveals selective, cell line-specific synergistic multidrug combinations

**Fig. 1:**
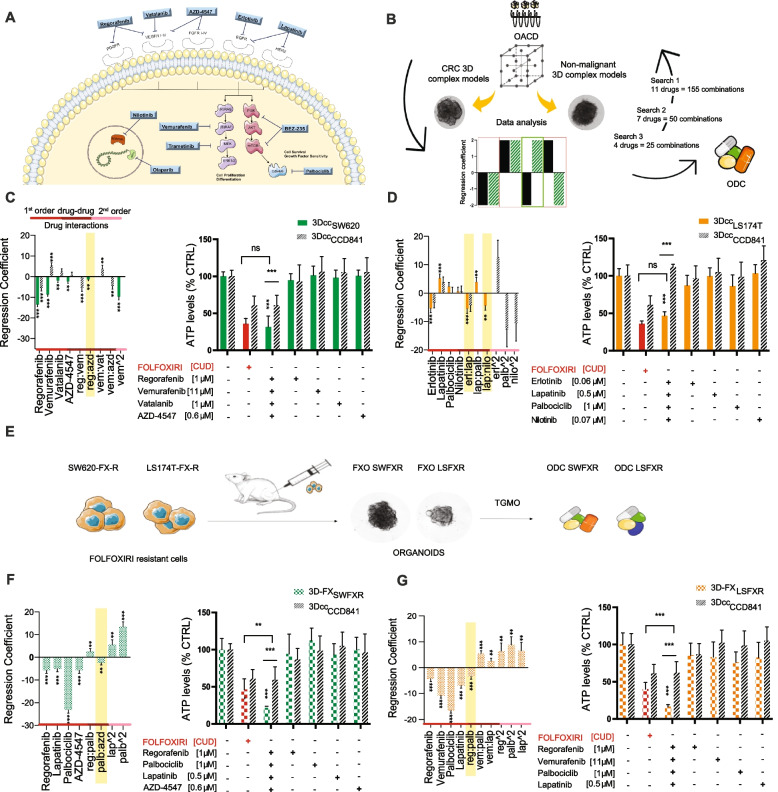
TGMO-based screen for cell line specific ODCs in CRC 3D complex models. **A** Initial selection of drugs used in the TGMO-based screen **B**. Schematic representation of the TGMO platform. Regression coefficients generated from search 3 of single drug 1^st^ order, drug-drug and single drug 2^nd^ order drug interactions (red, burgundy and pink lines, respectively) in **C **3Dcc_SW620_ **D **3Dcc_LS174T_ (green/orange bars respectively) and the therapeutic window (stripped black bars). **E** Schematic representation of the generation of complex CRC FOLFOXIRI resistant 3D models, 3D-FX_LSFXR_ and 3D-FX_SWFXR_ and respective ODC identification. **F** 3D-FX_SWFXR_ and **G** 3D-FX_LSFXR_ (solid green/orange squared bars respectively) in the left panel. In yellow is highlighted the most robust drug interaction that is maintained in each final ODC. In the corresponding right panels, the activity of the ODCs, corresponding monotherapies (colored bars) and FOLFOXIRI (folinic acid [0.5 µM], 5-FU [10 µM], SN38 [0.1 µM] and oxaliplatin [0.5 µM], red bars) in CRC 3D models, and activity in 3Dcc_CCD841_ (stripped black bars) used to generate the therapeutic window (TW). Activity is measured by ATP levels vs. CTRL (< 0.15% DMSO). Data are presented as the mean of *N* = 2–3 independent experiments, error bars represent SD. Significance is determined by one-way ANOVA (regression models, left panel) and two-way ANOVA (activity graphs, right panel) with *p < 0.05, **p < 0.01 and ***p < 0.001

To identify optimized multidrug combinations, we initiated our experiments with a set of 11 drugs, clinically approved or in late-phase clinical trials, see Fig. 1A and Table 1. To initiate the screen, drug-response curves for each drug were generated in two human CRC cell lines (SW620 and LS174T, Supplementary Table S1) in both, 2D and 3D co-cultures (3Dcc). The latter consisted of CRC cells, human colon CCD18co fibroblasts and ECRF24 endothelial cells [24]. Drug doses used in the screen corresponded to the IC_20_ and half of the latter. We kept the drug input low to only identify the strongest of drug-drug interactions. All drug concentrations selected were lower or equal to the clinically used dose (CUD), as indicated in Table 1. While for some drugs the dose–response curves in 2D overlapped with 3Dcc, for other drugs like palbociclib, vemurafenib and nilotinib, they were significantly different (Supplementary Figure S1-2).

**Table 1:** The set of drugs used in the screen, their main targets, and clinical information

Drug name	Main Target	CUD^a^ (µM)	Clinical phase	Clinical dose	AUC^b^ and reference
Regorafenib (Stivarga®)	VEGFR_2-3_, RET, PDGFR	4.3	approved	160 mg/kg	50.26 mg*h/L [25]; 58.3 mg*h/L [26]
Vemurafenib (Zelboraf®)	BRAF	32.3	approved	960 mg/kg	380 µg*h/mL (EMEA/H/C/002409)
Vatalanib	VEGFR_1-3_	6.3	Phase III	1200 mg	69.2 mg*h/L [27]; 52.9 mg*h/L [28]
Erlotinib (Tarceva®)	EGFR	1.6	approved	15 mg/kg	15.2 mg*h/L (EMEA/H/C/000618)
AZD-4547	FGFR_1-4_, VEGFR_2_	0.6	Phase II/III	160 mg	2058_(0-12 h)_ µg*h/L [29]
Lapatinib (Tyverb®)	HER2, EGFR	1.6	approved	1250 mg/kg	36.2 µg*h/mL (EMEA/H/C/002532)
Trametinib (Mekinist®)	MEK	14.4	approved	2 mg/kg	370 ng*h/mL (EMEA/H/C/002643)
Palbociclib (Ibrance®)	CDK_4-6_	29.3	approved	100/125 mg/kg	547.5 ng*h/L [30] (EMEA/H/C/003853)
BEZ-235	PI3K, mTOR	0.1	Phase II/III	400 mg/kg	741.3 ng*h/L [31]; 1404.4 µg*h/L [32]
Nilotinib (Tasigna®)	BCR/ABL	8.8	approved	300-400 mg	11,217 ng*h/mL [33] (EMEA/H/C/000798)
Olaparib (Lynparza®)	PARP	58	approved	400 mg	609 µg*h/mL [34] (EMEA/H/C/003726)

^a^*CUD* Clinically used dose, calculated using the area under the curve

^b^(AUC_0-24 h_) of each drug, which corresponds to the plasma concentration of the drug over the first 24 h, and is used to calculate the average drug concentration in that time. Data was obtained from pharmacokinetic studies performed in patients exposed to the drugs at standard or maximum tolerated doses

Using our validated phenotypical screening method, the TGMO (see Supplementary Information S1), we tested several drug combinations in 3Dcc settings for both—non-malignant colon CCD841CoN cells (CCD841), and simultaneously in CRC cells. The difference between the activity in non-cancerous and cancerous models is defined as the therapeutic window (TW) and was used to evaluate the selectivity of each drug combination. The output was measured by a cell metabolic activity assay (ATP level, % CTRL) and analyzed using stepwise second-order linear regression model**.** Based on the analysis, drugs with antagonistic interactions were eliminated, and the next screening round was performed (search 2), Fig. 1B. The regression models generated from each search are presented in Supplementary Figs. S3-4. The last search 3 served to optimize the drug doses within the selected drug combinations (Fig. 1C-D). The models allow to describe the different interactions present in the drug pool: the contribution of each drug individually (single drug first-order term), all two-drug interactions, and the variation of a drug-effect at different dose levels (single drug second-order terms). The optimized drug combination (ODC) for each cell line consisted of four active and synergistic drugs administered at specific doses. ODC_SW620_ consisted of regorafenib [1 µM], vemurafenib [11 µM], vatalanib [1 µM] and AZD4547 [0.6 µM], inhibiting 68.5% of 3Dcc_SW620_ metabolic activity and 39.2% in 3Dcc_CCD841_ (Fig. 1C). ODC_LS174T_ consisted of erlotinib [0.6 µM], lapatinib [0.5 µM], palbociclib [1 µM] and nilotinib [0.7 µM] and inhibited 53.3% of 3Dcc_LS174T_ while being inactive in 3Dcc_CCD841_ (Fig. 1D). In all conditions, the activity of ODCs significantly outperformed the corresponding monotherapies. Interestingly, no common drugs appeared in the final selection of both ODCs. The main synergistic drug interactions within the ODCs are highlighted in yellow in the regression models presented in Fig. 1-CD, left panels, and listed in Table 2 and Supplementary Table S1.

**Table 2:** Drug-drug interactions and combination index (CI) of ODCs in 3D models

		**ODC**_**SW620**_	**ODC**_**LS174T**_	**ODC**_**SWFXR**_	**ODC**_**LSFXR**_
TGMO	Synergisms	reg:azd	erl:lap lap:nilo	palb:azd	reg:palb
CI	ODC	0.42	0.11	0.006	0.27

Drugs: reg, regorafenib, azd, AZD-4547; erl, erlotinib; lap, lapatinib; palb, palbociclib; nilo, nilotinib. CI < 1: synergistic drug combination, CI > 1: antagonistic drug combination

In the next step, we searched for the ODCs identified in CRC cells, that were chronically treated with chemotherapy. This was done to mimic the clinically relevant situation where patients with advanced CRC are treated with combinatory chemotherapy. To that end, FOLFOXIRI-resistant clones of LS174T and SW620 cells previously generated in our lab [35], were injected subcutaneously into Swiss nude mice. When tumors reached an appropriate size, they were resected and two distinct complex CRC FOLFOXIRI resistant 3D models (3D-FX_LSFXR_ and 3D-FX_SWFXR_, respectively) were created. To extend our search for ODCs, we performed a TGMO-based screen on both organoids. Data analysis and regression models are presented in Supplementary Fig. S5. The identified ODCs are referred to as ODC_SWFXR_ and ODC_LSFXR_. The ODC_SWFXR_ consisted of regorafenib [1 µM], palbociclib [1 µM], lapatinib [0.5  M] and AZD-4547 [0.6 µM], inhibiting 78.6% of FXO_SWFXR_ metabolic activity (Fig. 1F). ODC_LSFXR_ was composed of regorafenib [1 µM], vemurafenib [11 µM], palbociclib [1 µM] and lapatinib [0.5 µM], inhibiting 83.9% of FXO_LSFXR_ metabolic activity (Fig. 1G). Both ODCs showed significantly lower activity in 3Dcc_CCD841_.

The activity of the ODCs was then compared to drugs approved in clinics as first-line treatment for late- stage CRC. Moreover, all four ODCs showed similar or significantly higher cytotoxic effects than FOLFOXIRI (folinic acid [0.5 µM], 5-FU [10 µM], SN38 [0.1 µM] and oxaliplatin [0.5 µM]) given at clinically used doses.

### TGMO-based sensitivity analysis in the view of penalized regression

**Fig. 2:**
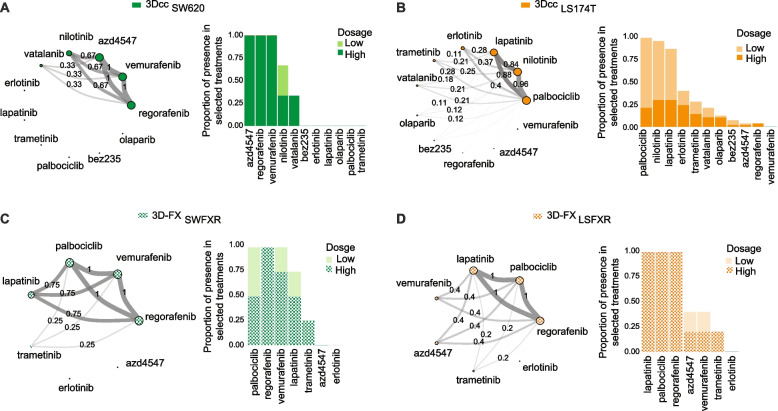
ODC search using adaptive lasso. Left panel represents the network diagram of the drugs composing best ODCs with an PTW that falls into the CCI (95%) in 3Dcc _SW620_ (**A**) and 3Dcc _LS174T_ (**B**), or with a minimal cancer cell viability that falls into the CCI (95%) in 3D-FX_SWFXR_ (**C**) and 3D-FX_LSFXR_ (**D**). In the corresponding right panel, we considered the dosages of each drug using two colors stacked bar graphs, where the height of a bar is proportional to the presence of the drug at a given dosage in all combinations in the CCI

We investigated further our findings obtained with the TGMO method by performing a sensitivity analysis. We applied a penalized regression approach, described in Supplementary Information S2, on our raw data obtained from TGMO-based design of experimentation. We predicted the cell viability of cancer cells and healthy cells with a data-dependent adaptive version of the Lasso to identify all possible combinations of four drugs at applied dosages [36]. This method allows us to approximate the set of non-distinguishable combinations of drugs. Based on the predicted cell viability, we computed the predicted therapeutic window (PTW) for each drug administered at a specific dose. We then constructed a one-sided confidence interval (95%) based on the highest PTW and considered all combinations of drugs with an undistinguishable PTW that fall into this interval. When a TW is not available (i.e., 3D-FX_SWFXR_ and 3D-FX_LSFXR_) a computed one-sided confidence interval (CCI, 95%) was generated based on the minimum value of predicted cancer cell viability, and all drug combinations with a predicted cancer cell viability within this interval were selected. The generated regression models are presented by network graphs and stacked bar graphs, see Fig. 2. The size of the node associated with each drug in the network graphs is proportional to the presence of a given drug in all combinations within the CCI. The thickness of the link between two drugs is proportional to the presence of this pair of drugs in all combinations within the CCI. Using the data-dependent adaptive lasso approach, we identified 3 and 57 drug combinations (in 3Dcc_SW620_ and 3Dcc_LS174T_ models, respectively) in the CCI computed on the maximal PTW of 5280 possible combinations. In 3Dcc_SW620_ model (Supplementary Table S3), regorafenib, vemurafenib, AZD-4547, nilotinib and vatalanib were the most present drugs, with all three identified combinations being composed of regorafenib, vemurafenib and AZD-4547 at the high dosage, see Fig. 2A. For 3Dcc_LS174T_ model, 14 out of the 57 drug combinations, (Supplementary Table S4) combinations consisted of the same four drugs, i.e., palbociclib, nilotinib, lapatinib and erlotinib (Fig. 2B). On the stacked bar graphs, these drugs were mostly administered at low doses. Interestingly, both ODC_SW620_ and ODC_LS174T_ identified by the TGMO were present in their respective CCI. Furthermore, we identified 4 and 5 drug combinations in 3D-FX_SWFXR_ and 3D-FX_LSFXR_, respectively, in the CCI computed on the minimum value of predicted cancer cell viability of 560 possible drug combinations. For 3D-FX_SWFXR_, three out of four of the selected drug combinations, (Supplementary Table S5) were composed of lapatinib, palbociclib, regorafenib and vemurafenib at different doses (Fig. 2C), while all five selected drug combinations in 3D-FX_LSFXR_ (Supplementary Table S6) were composed of palbociclib, regorafenib and lapatinib administered at high dosage, see Fig. 2D. The ODC_SWFXR_ identified by the TGMO, consisting of regorafenib, lapatinib, palbociclib and AZD-4547, was not recovered in the CCI, while ODC_LSFXR_ containing regorafenib, vemurafenib, palbociclib and lapatinib was ranked first in their corresponding CCI.

### ODCs activity is confirmed in patient-derived organoids

**Fig. 3:**
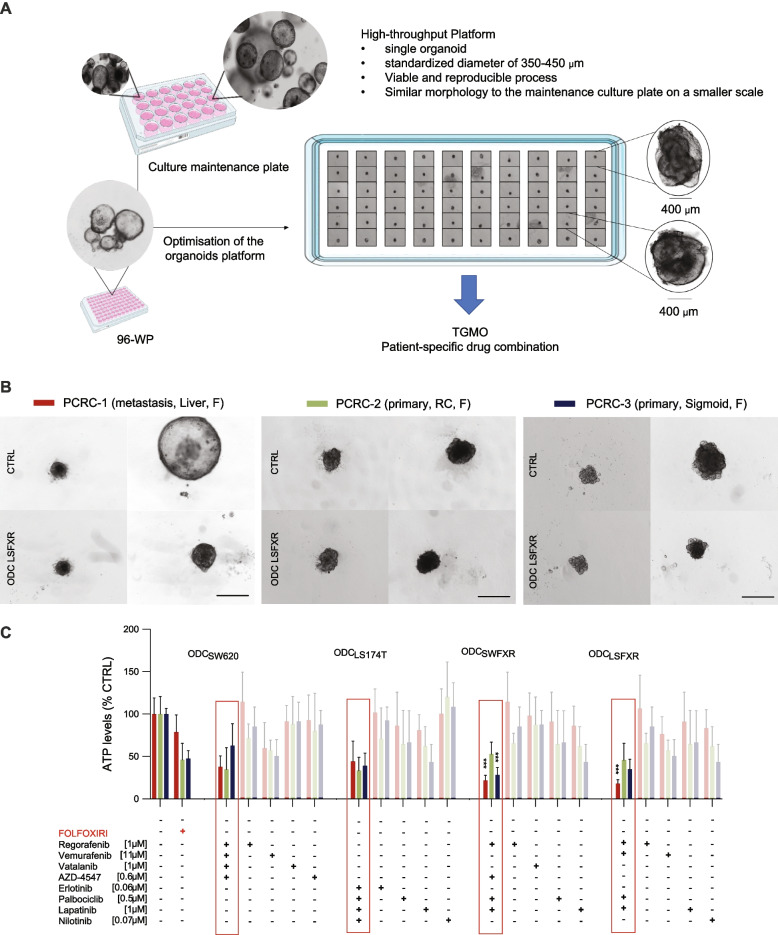
Cross-validation of ODCs activity on patient-derived organoids. **A** Schematic representation of our patient-derived organoids platform. **B** Representative images of PDOs from PCRC-1, PCRC-2 and PCRC-3 treated with ODC_LSFXR_. Scale bar represents 500 µm **C**. Activity of all four ODCs, and corresponding monotherapies and FOLFOXIRI (folinic acid [0.5 µM], 5-FU [10 µM], SN38 [0.1 µM] and oxaliplatin [0.5 µM], red bars) in PDOs from PCRC-1 (burgundy bars), PCRC-2 (dark green bars) and PCRC-3 (navy bars). Activity is measured by ATP levels vs. CTRL (< 0.15% DMSO). Data is presented as mean of *N* = 3 independent experiments, error bars represent SD. Significance is determined by two-way ANOVA with *p < 0.05, **p < 0.01 and ***p < 0.001

To validate the activity of the cell line specific ODCs, we exposed freshly isolated patient-derived organoids to those ODCs for 72 h, see Fig. 3. The PDOs originated from three different patients, i.e., a patient presenting liver metastasis (PCRC-1, burgundy bar), a patient with right colon primary tumor (PCRC-2, dark green bar), and a patient with sigmoidal primary tumor (PCRC-3, navy bar), Fig. 3A. The PDOs were passaged in standard culture conditions. Subsequently, organoids were collected, dissociated, counted, and plated at the desired density. When single organoids reached a diameter of approximately 350–400 μm, commonly accepted size to mimic conditions where necrotic core may appear [37, 38], they were exposed to the therapy of choice for 72 h. In all ODC_LSFXR_-treated PDOs, the decrease in their size and change in morphology was observed, Fig. 3B. However, since we previously showed that size might not be an exact delimitator of drug activity in 3D cultures [24], thus the ATP levels were measured and compared between the conditions. In addition, ODC_LSFXR_ activity in the PDOs was confirmed using H&E staining (Supplementary Information S4 and Supplementary Fig. S7). A clear decrease in tumors size and structure is noticeable in the ODC-treated group. All ODCs (see the ODCs composition in Fig. 1) were active in three types of patient-derived organoids, as compared to the corresponding monotherapies and to FOLFOXIRI, which was used as a positive control, see Fig. 3C. Interestingly, ODC_SWFXR_ and ODC_LSFXR_, of which composition differed only by one drug, seemed to be highly potent in PCRC-1 and PCRC-3. Whereas all three PCRCs were prone to vemurafenib, and unsensitive to vatalanib, AZD-4547 or nilotinib, their sensitivity to other monotherapies differed.

### Gene expression profiles and genomic mutations landscape in PCRC organoids

**Fig. 4:**
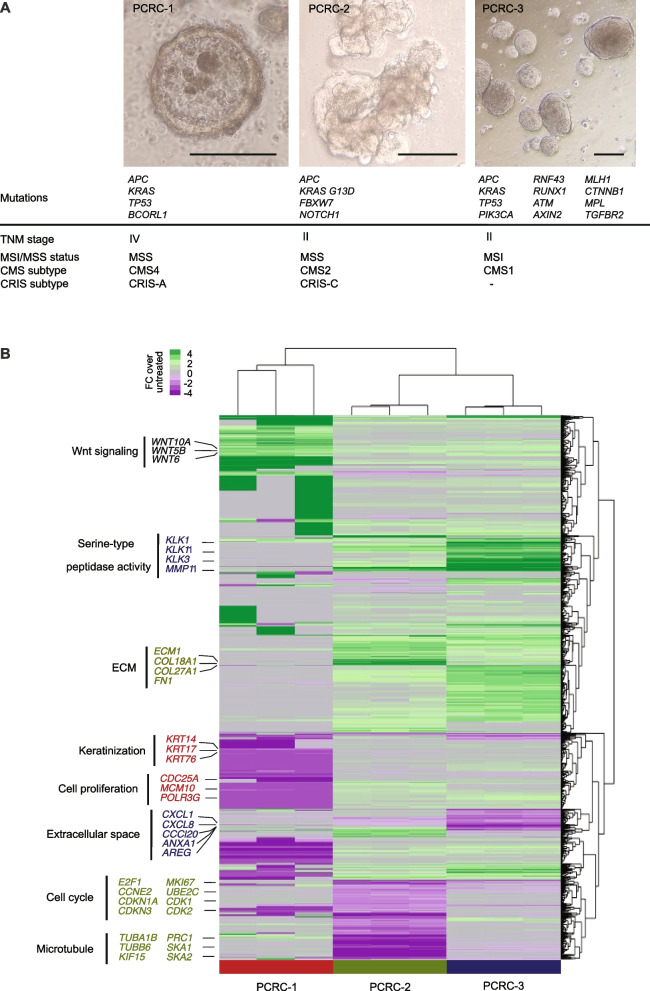
Gene expression profiles and genomic mutations landscape in PDOs. **A** Representative images of the PDOs established from patients PCRC-1–3, their mutations in known CRC-related genes, and TNM stage, CMS, CRIS and MSS/MSI classification. Scale bars represent 200 μm, **B**. Gene expression changes of established PDOs from PCRC1-3 after treatment with ODC_LSFXR_ for 72 h (*N* = 3). Differentially expressed pathways (Genes with the |log2FC|> 2) are highlighted in the right panel

Cellular phenotype and mutation landscape determine drug sensitivity. Whole-exome sequencing (WES) of the PDOs (Fig. 4) revealed that PCRC-1 presented alterations in common CRC mutated genes, such as *APC*, *KRAS* and *TP53*. PCRC-2 contained alterations in *APC*, *KRAS*, *FBXW7* and *NOTCH1* genes, Fig. 4A. PCRC-3 had an inactivating mutation in *MLH1* causing deficiency in MMR. Indeed, PCRC-3 had 3–4 times more mutations than the other patients including mutations implicated in CRC: *APC*, *KRAS*, *TP53*, *ATM*, *CTNNB1*, *AXIN2*, *RNF43*, *PIK3CA*, and *RUNX1*.

Established PDOs were also sequenced using RNA-seq and gene expression profiles of untreated samples were used to establish the classification of the patients according to the CMS/CRIS schemes and MSS status [7, 8, 39], Fig. 4A. PCRC-2 was classified as CMS2/CRIS-C and MSS, which correlated with the histopathological evaluation of the patient's tumor. The CMS2/CRIS-C signatures describe the classical Wnt^high^ cohort of tumor samples. Indeed, PCRC-2 has mutations in tumor suppressor genes *APC* and *FBXW7*. FBXW7, like APC, antagonizes Wnt activity by targeting β-catenin for degradation [40]. PCRC-3 was classified as MSI high, CMS1 subtype of CRC, which correlates with the MLH1 mutational status and high number of SNPs. PCRC-1 was classified as CMS4/CRIS-A. The above classification confirmed that our cohort of samples covered very distinct CRC sub-groups.

We have further assessed gene expression changes upon treatment of all three PDOs with ODC_LSFXR_ for 72 h Fig. 4B. Hundreds of genes were differentially expressed in each PDO upon treatment. Interestingly, relatively few genes were similarly deregulated in all PDOs including several Wnt-signaling related genes (*WNT10A*, *WNT5B*, *WNT6*). PCRC-1 showed downregulation of keratinization and cell proliferation-related genes upon treatment. PCRC-2 showed up-regulated extracellular matrix (ECM) proteins, such as *ECM1*, *FN1*, *COL18A1*, and *COL27A1*. At the same time, cell cycle-related genes (*CCNE2*, *MKI67*, *CDK1/2*, *E2F1* and others) and genes important for microtubule formation (*TUBA1B*, *TUBB6*, *SKA1/2*, *KLF15*) were downregulated. PCRC-3 unlike PCRC-2, showed a down-regulation of ECM-related genes but showed an enrichment in genes involved in serine-type peptidase activity (*MMP11*, *KLK1/3/11*). Two PDOs showed a significant reduction in cell proliferation upon treatment indicating the effective action of the ODC_LSFXR_ drug combination.

Together, this data show that PDOs have distinct mutational status, CMS/CRIS/MSS classification and heterogenous gene expression responses. This supports the concept that each patient tumor would eventually require a unique personalized drug combination for optimal treatment response.

### Identification of synergistic multidrug combinations directly in patient-derived organoids (PDO)

**Fig. 5:**
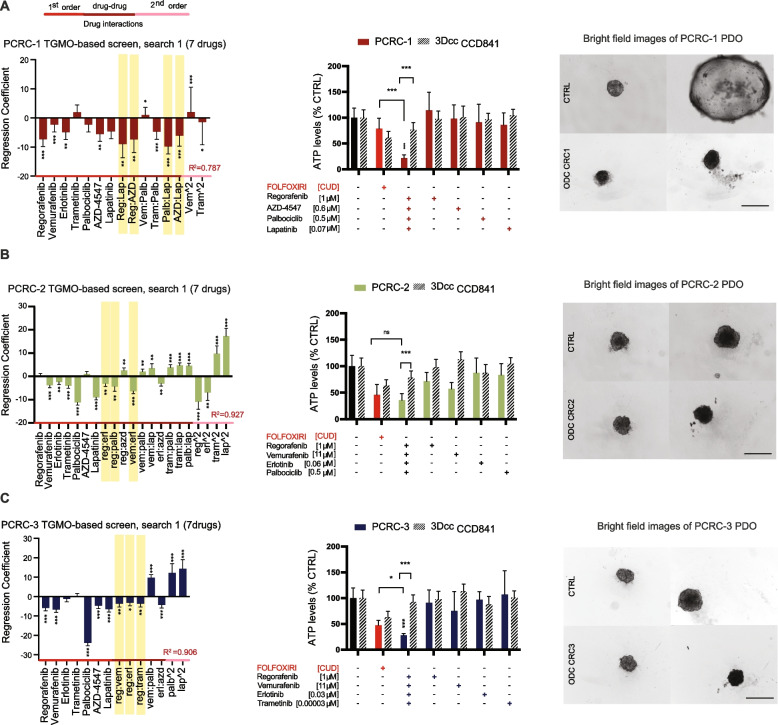
Optimization of patient specific ODCs. **A** Regression coefficients generated from search 1 of the TGMO-based screen on PDOs, describing single drug 1^st^ order, drug-drug and single drug 2^nd^ order drug-drug interactions (red, burgundy and pink lines, respectively) for CRC-1 **B**. CRC-2 and **C**. CRC-3 (left panels). In yellow is highlighted the most robust drug-drug interaction in each patient specific ODC. In the corresponding middle panels, activity of the patient specific ODCs, corresponding monotherapies (solid colored bars), and FOLFOXIRI (folinic acid [0.5 µM], 5-FU [10 µM], SN38 [0.1 µM] and oxaliplatin [0.5 µM], red bars) in each PDO and in CCD841 3Dcc (black stripped bars). Activity is measured by ATP levels vs. CTRL (< 0.15% DMSO). Data is presented as mean of *N* = 3 independent experiments, error bars represent SD. Significance is determined by one-way ANOVA (regression models, left panel) and two-way ANOVA (activity graphs, right panel) with *p < 0.05, **p < 0.01 and ***p < 0.001. In the right panels, representative images of PDO treated with their corresponding patient specific ODC. Scale bar represents 500 µm

As shown above, the ODCs optimized in 3Dcc models based on CRC cell lines were also quite active in PDOs, Fig. 3. To know how representative those ODCs are, we performed a TGMO-based screen directly on the same PDOs, i.e., PCRC-1, -2 and -3 (Fig. 5 right panels with representative organoid bright field images). To maintain the same quality of freshly isolated patient material and to maintain a clinically relevant timeframe, we performed only search 1 on PDOs, which consisted in screening 50 different drug combinations from our seven drug-pool, each given at two doses, followed by data modeling. We narrowed the search as compared to the initial set of eleven drugs based on the most effective/safe seven drugs in the 3Dcc models. The regression models generated (Fig. 5, left panels), showcased the different interactions present in the drug pool. In yellow we highlighted the most robust drug interactions that were included in each final ODC. Interestingly, ODC_PCRC-1_ (Fig. 5A), ODC_PCRC-2_ (Fig. 5B), and ODC_PCRC-3_ (Fig. 5C) were composed of different drugs and were slightly more active than the ODC identified in the cell lines (ODC_LSFXR_). ODC_PCRC-1_ consisted of the same drugs as ODC_SWFXR_. The ODCs inhibited 78.1%, 64.2% and 72.3% of PDOs cell viability in PCRC-1,-2,-3, respectively, while being significantly more active than FOLFORIXI in case of PCRC-1 and -3 (Fig. 5B).

## Discussion

Optimizing low-dose synergistic drug combinations is an attractive strategy to tackle the complex machinery of cancer and to overcome, or more importantly, to prevent acquired drug resistance. The simultaneous targeting of key signaling pathways at different levels or targeting distinct signaling pathways is essential to efficiently kill cancer cells.

Using our validated phenotypically driven platform (TGMO), we have previously identified synergistic optimized drug combinations in a panel of CRC cell lines in 2D cell models [21]. In this study, we further emphasized the importance of this strategy in a more complex clinically relevant models, notably patient-derived organoids, that recapitulate more faithfully the tumor organization and micro-environment. Moreover, we extended our statistical analysis pipeline to include the adaptive lasso approach, which strengthened the analysis performed by the TGMO.

Our results show a broad activity of ODC_LSFXR_, consisting of low dose regorafenib (VEGFR inhibitor), vemurafenib (BRAF inhibitor), lapatinib (EGFR and HER2 inhibitor) and palbociclib (CDK4/CDK6 inhibitor), in 3D co-culture*s* (Supplementary Fig. S6), and with PDOs, notably in PCRC-1, which was resistant to chemotherapy treatment (Fig. 3). This can be explained by the inhibition of four different key signalling pathways in CRC both upstream and downstream in the cell. This is in line with the findings of Neto et al*.* who recently reported that inhibition of RAF/MEK/ERK with upstream inhibition of EGFR using 3 to 4 drugs in combination was needed to completely block oncogenic activated MAPK signaling, and could overcome acquired resistance to high dose monotherapy [41]. In the study of Horn et al*.,* drugs targeting PI3K/AKT/mTOR and MAPK signaling pathways combined with agents targeting CDK4/6 family protein were identified in their most optimal drug combination against colorectal cancer [42]. The efficacy of such drug mixtures was also validated in other cancer types with similar key driving mutations. The simultaneous dual inhibition of these pathways was validated in advanced stage ovarian cancer, where upon treatment with a low dose synergetic triple combination, significant reduction in tumor size in patient-derived xenograft models was observed [43].

Our results show that the effect of the drugs differs between 2 and 3D models, see Supplementary Figs. S1-S2. Even though 3D models are more complex, we observed a reduction in the concentration of some drugs like regorafenib and vemurafenib in the ODC identified in this study, when compared to the ODCs previously optimized in 2D models [21]. Our findings converge with the recent study of Folkesson et al*.*, where this reduction in concentration is due to higher synergistic effects that have been observed in CRC 3D models compared to 2D, especially when treated with combinations including MEK inhibitors [44]. This is also in line with our previous findings, where we observed a significant reduction of the dose of erlotinib in both homogenous and heterotypic CRC 3D models compared to 2D cell models [24].

HER2 has been recently considered an emerging biomarker in CRC, especially in the metastatic settings. HER2 overexpression/amplification was reported in 3–5% of metastatic CRC [45]. Multiple phase II clinical trials (HERACLES) [46] have shown that HER2 blockade was beneficial for (patients with metastatic CRC refractory to chemotherapy) [47]. In our TGMO-based screen, lapatinib, a reversible EGFR and HER2 inhibitor, figured in the optimal ODC identified on organoids 3D-FX_SWFXR_ and 3D-FX_LSFXR_. Moreover, the strength of the lapatinib effect was correlated with the overall HER2 expression in PDOs. PCRC-2 had tenfold higher expression of HER2 compared to the other patients, at the same time being the most vulnerable to the lapatinib treatment. Furthermore, it has been shown that CDK4/6 inhibition potentiates the effect of anti-HER2 drugs. In fact, cyclin D1 as a resistance pathway, and studies have shown that the inhibition of CDK4/6 downstream simultaneously while targeting upstream the cells can potentiate the efficacy of treatment [48]. These findings converge with the results of our drug screen, where lapatinib was always identified in tandem with palbociclib. The frequency of simultaneous occurrence of both drugs in our search was also validated using adaptive lasso, where the mathematical model always linked palbociclib to lapatinib in FOLFOXIRI-resistant models (FXO_SWFXR_, FXO_LSFXR_ and PCRC-1). Furthermore, ODC_PCRC-1_ consisted of regorafenib [1 μM], palbociclib [1 μM], lapatinib [0.5 μM] and AZD-4547 [0.6 μM], the same drug composition and drug doses as for ODC_SWFXR_. In fact, the mutational profile of PCRC-1 (Fig. 3), has mainly the same three key mutation implicated in CRC as the SW620 cell line (*KRAS, APC* and *TP53*), see Supplementary Table S2. PCRC-1 was classified as a CMS4, a subtype usually identified for stage IV tumors and linked to lowest overall survival [7], but was efficiently treated with ODC_PCRC-1_. This ODC showed in our models 3.6-fold higher activity than FOLFOXIRI administered at CUD (Fig. 5A).

PCRC-2 was classified as CMS2 and CRIS-C, the latter known to have highly elevated EGFR signaling and therefore sensitive to EGFR inhibition. PCRC-2 also contained a mutation in *KRAS,* which is often associated with resistance to EGFR-targeted therapy. However, studies have shown that patients with the *KRAS G13D* mutation, as in PCRC-2, are an exception to this dogma and respond to the therapy [49–51]. This has been argued by others, e.g. Rowland *et.al*, who stated according to their meta-analysis, that there is no significant difference in response to anti-EGFR therapy between *KRAS G13D* and other *KRAS* mutants with metastatic CRC [52, 53]. The exact mechanism behind this unusual behavior of *KRAS G13D* mutants is yet to be fully elucidated [54].

This is the first time we apply our phenotypic TGMO approach directly on patient-derived material withing the clinically relevant timeframe. We carefully showcase that there exists a rationale behind each ODC, and that each drug combination is different in terms of drugs and doses according to the specific mutations and gene expression programs of each corresponding PDOs. Even though ODC_LSFXR_ is very potent and is active in all PDOs, its activity remains undistinguishable compared to the ODCs identified specifically for each patient (Fig. 3*vs.* Fig. 5).

Moreover, the sensitivity analysis conducted based on adaptive regression techniques is in line with the results of the TGMO. This alternative method demonstrates the reliability of the TGMO, while pointing towards potential improvements in terms of experimental burden. Both approaches are based on different underlying assumptions and consequently have different strength and limitations. However, their methodological comparison is beyond the scope of this study and is left for further research.

It is also important to mention that in this study we eagerly contributed to the development of alternative to animal use models with respect to the 3Rs for replacement, reduction and refinement. We show that advanced but easy-to-use 3D assays can be predictive in terms of drug activity and significantly reduce the number of animals needed to bring new cancer treatment to the clinical use.

There are some bottlenecks that we have identified and that could be improved in further experiments. First, so far, we failed in the isolation of healthy colon epithelial organoids from the same patients. This is a frequent problem also recognized by other research groups, as the rate of non-cancerous organoid establishment is very low, approx. 6% of the cells that could be passaged [55]. We used complex 3D co-cultures (Fig. 1) to validate the safety of our ODCs, while recognizing the fact that they do not fully represent a proper intra-patient control. We are currently working on optimizing our isolation and maintenance protocol to secure experiments in the non-cancerous organoids for each patient.

Another point is the choice of a phenotype as a proxy for the activity of the different ODCs, i.e., metabolic cell activity in our case. To distinguish long-term superiority between the selected drug combinations, we plan to consider additional phenotypes and longer treatment periods in future experiments. In addition, the different drugs in the pool target some of the most important genes and pathways currently known in cancer pathogenesis. Although each compound was selected for a particular target (i.e. Vemurafenib – *BRAF)*, most inhibits several other targets (i.e. ragorafenib: VEGFR1-3, TIE2, PDGFR-β, FGFR, KIT, RET, and RAF) [56]. Therefore, it is also possible that the activity of our compound mixture can be related to some “minor target” inhibition by one compound or the common inhibition of a “minor target” by several compounds. Testing this hypothesis is outside the scope of this paper and will require further in vitro work. In addition, since CRC is known to be genetically diverse and may be different within the intratumor geography [57], in future studies we will compare the different genetic clones of organoids in their response to treatments.

Thirdly, we are working on the elaboration of toxicity and pharmacological activity scales of the ODCs. Defining toxicant mixture composition with the aim to map metabolic networks is a delicate task, as they should present different mechanisms of action that would in turn produce specific metabolic phenotypes. We think that by phenotyping the ODCs, administered at low doses, we will be able to explore highly diverse metabolic responses and thus evidence more complete metabolic subnetworks relevant to the identification of main key-events relationships. We hypothesize that these underlying metabolic networks play a significant role in understanding toxicology-related health problems.

## Conclusion

Summarizing, our study represents an innovative experimental PDO-platform, which combined with streamlined in silico data analysis, may allow optimization of synergistic multi-drug combination therapy tailored specifically to individual patients and stratified patients to selected treatments in a clinically relevant timeframe.

## Materials and methods

### Cells and culture conditions

LS174T and SW620 human CRC, as well as CCD18co and CCD841 non-malignant cells were obtained from ATCC with a corresponding authentication certificate. Human immortalized endothelial cells ECRF24 cells (via immortalization procedures with amphitropic replicant-deficient retrovirus [58]). LS174T and SW620 FOLFOXIRI resistant cell lines were generated in our laboratory through a chronic treatment [14, 35]. The cells were cultured in a humidified incubator at 37 °C and 5% CO_2_ in culture medium supplemented with 10% fetal bovine serum (S1810-500, Biowest, Nuaillé, France) and 1% penicillin/streptomycin (4-01F00-H, BioConcept, Allschwil, Switzerland). Cells were regularly tested for mycoplasma contamination using the MycoAlert kit (LT07-218, Lonza, Rockland, ME, USA), with controlled passage.

3D co-cultures were created in 96-well U-bottom low attachment plates (650970, Greiner Bio-One, Frickenhausen, Germany) with CRC cells or CCD841 cells seeded 1:1 with CCD18co cells and 5% ECRF24 cells [24]. Culture media consisted of a mixture of DMEM, RPMI and EMEM (1:1:1) supplemented with 2.5% Matrigel® (354254, Corning, Bedford, MA, USA). The 3Dcc were treated 48 h post-seeding (Day2).

### Tumor isolation and organoids establishment

Colorectal cancer samples were obtained from University Hospital in Geneva (HUG). The study methodology with the use of patient-derived material was approved by the Swiss Ethics Committee on research involving humans (2017–00364) and conformed to the standards set by the Declaration of Helsinki. The experiments were performed with a written consent from each patient.

The patient-derived CRC tissue was transported in DMEM-F12 (10565–018, Gibco) and 1 × Primocin (ant-pm-1, Invitrogen, Toulouse, France), and processed within approx. 1 h after resection. Tissues were washed with HBSS (14170–088, Gibco) and mechanically dissociated into 1–2 mm^3^ cubes with a surgical blade in a small glass petri dish in 1 mL digestion medium (DMEM/F12 + Liberase DH (0.28unit/ml)). Tumor pieces were then transferred to GentleMACS C tubes (5171215296, Miltenyi Biotec, Germany) and enzymatically digested for 1 h. The samples were then filtered, and the retained fragments were collected and washed twice in HBSS. The washed samples were then resuspended in the adequate volume of Matrigel® and incubated in a 5% CO_2_-humidified incubator at 37 °C for 20 min. Then serum-free stem cell medium DMEM/F12 + StemPro hESC (A10006-01, Gibco) supplemented with 8 ng/mL hFGF. See more detailed protocol under Supplementary Information S3.

### Drugs and treatments

Drugs were dissolved in DMSO to prepare stock solutions at the corresponding concentrations. Aliquots were stored at -80 °C and thawn prior to each experiment. Regorafenib (R-8024, 20 mg/mL), vemurafenib (V-2800, 50 mg/mL), erlotinib (E-007, 15 mg/mL), lapatinib (L-4904, 20 mg/mL), Palbociclib (P-7744, 30 mg/mL),, trametinib (T-8188, 15 mg/mL), nilotinib (N-8207,10 mg/mL), olaparib (O-920, 10 mg/mL), BEZ-235 (N-4288, 10 mg/mL) were purchased from LC labs (Woburn, MA, USA); vatalanib (PTK787, 10 mg/mL) from SelleckChem (Houston, Texas, USA); AZD-4547 (HY-13330,10 mg/mL) and SN38 (HY-13704/CS-1579, 1 mg/mL) from MedChemExpress (Monmouth Junction, New Jersey, USA); 5-flurouracil (F6627, 10 mg/mL), folinic acid (F7878, 20 mg/mL), oxaliplatin (O9512, 5 mg/mL in UltraPure distilled water) from Sigma-Aldrich. Cells were exposed to single drugs or pre-mixed drug combinations for 72 h for 2D, 3Dcc and PDOs. Corresponding cell culture medium with and without 0.15% DMSO was used as control.

### Metabolic ATP activity assays

Drug treatment activity was measured using the 3D CellTiter-Glo® cell metabolic activity (ATP) assays (G9683, Promega, Madison, WI, USA), according to the manufacturer`s instructions. Assay bioluminescence was detected using the BioTek Cytation 3 and corresponding Gen5 Image software version 3.04 at standard settings.

### Combination index (CI) calculation

The combination index of the different drug combinations was calculated using the software Compusyn. The input consists in the fraction affected (FA) calculated as follows: 1 – (raw ATP levels of treated condition / raw ATP levels of control).

### mRNA transcriptome and analysis

The libraries (for PCRC-1 untreated and treated samples) were prepared using MERCURIUS BRB-seq kit (ALITHEAgenomics) according to the manufacturer protocol, using 15 cycles for the libraries amplification. The libraries were sequenced on NovaSeq 6000 using next sequencing structure: read1:i7 index:i5 index:read2 = 28:8:8:90. Gene expression profiles were normalized across the samples based on the number of total counts (25 mio counts/sample). Lowly expressed genes (~ 20 counts/sample) were removed. RNA easy® Plus Kit (74134, Qiagen, Hilden, Germany) was used to extract RNA from PDOs of PCRC-2 and PCRC-3 according to the manufacturer’s instructions. RNA quantification was performed with a Qubit fluorimeter (ThermoFisher Scientific) and RNA integrity assessed with a Bioanalyzer (Agilent Technologies). The TruSeq mRNA stranded kit from Illumina was used for the library preparation with 150 ng of total RNA as input. Library molarity and quality were assessed with the Qubit and Tapestation (DNA High sensitivity chip). Libraries were sequenced on a NovaSeq 6000 Illumina sequencer for SR100 reads.

Genes which were differentially expressed (|log2FC|> 2) between control and treated samples were selected for each patient. 3 sets of genes (for 3 patients) were combined and plotted using heatmap package in R.

### Whole exome sequencing (WES) and analysis

For PCRC-1, mutation profile is routinely performed by clinical pathology (HUG). DNA was extracted from two FFPE tissue sections of about 10 μm with Promega Maxwell® RSC DNA FFPE Kit (Promega AS1450) on the automated machine (Promega RSC). The concentration was measured using fluorometric Qubit reagents (Thermofisher Scientific, cat n° Q32851). 50 ng DNA was used to generate a sequencing library with a custom 100-gene panel using the SureSelect XT-HS Target Enrichement System from Agilent Technologies (Santa Clara, USA). The sequencing was performed on an Illumina NextSeq 500 sequencing system. The results were analysed using an in-house analysis software and variant calling performed with Oncobench® (Swiss Institute of Bioinformatics).

For PCR2-3, organoids were collected, trypsinized to single cells and resuspended in 200 μL of PBS. DNA was isolated from the corresponding samples using NucleoSpin Blood, Mini kit for DNA from blood (Macherey–Nagel, REF: 740951.50) according to the manufacture protocol. Exome capture was performed using xGen Exome Research Panel v2 (IDT, REF: 10005152), xGen Hybridization and Wash Kit IDT (ref 1080577), xGen Universal Blockers-TS IDT (ref 1075474). Library preparations were done using xGen DNA Library Prep EZ kit (REF: 10009863). Amplified libraries were sequenced using NovaSeq SP PE configuration at 150 cycles at 84 × sequencing depth (20 million reads per sample). Obtained fastq files were trimmed with TrimGalore (version 0.6.6) using the standard settings (adapter sequence 5’- AGATCGGAAGAGC-3’). The alignment was done with BWA mem (BWA version 0.7.17) using Homo_sapiens.GRCh38.dna.primary_assembly as a reference genome. Samtools (version 1.10) was used to obtain corresponding bam files. Picard (version 2.20.8) and GATK (version 4.3.0.0) were used to prepare and filter the bam files using MarkDuplicates, AddOrReplaceReadGroups, BaseRecalibrator (NCBI library of known sites were used common_all_20180418.vcf.gz as a reference), ApplyBQSR functions. GATK was used to call haplotypes and obtain corresponding vcf files using HaplotypeCaller function, as well as for consolidating corresponding vcf files, selecting SNP and INDEL variants and variant filtration. snpEff was used for variants annotation for human genome (GRCh38.86). GATK VariantsToTable function was used to obtain the final table containing all SNPs and INDELs. ClinVar database of pathogenic variants and OncoKB database of known variants for 500 most mutated genes in CRC were used to select the corresponding relevant variants.

### Subcutaneous in vivo tumor models

Subcutaneous implantation of LS174T-FX-R and SW620-FX-R was performed as described previously [21]. In short, 6–8 week-old male nude mice were implanted with cell suspensions of 50 μL serum free medium containing 10 μg/uL Matrigel® with 5000 cells/mouse for both LS174T-FX-R and SW620-FX-R [59], subcutaneously in the right flank. When tumor size reached 1000 mm^3^, after 7 days and 15 days (LS174T-FX-R and SW620-FX-R, respectively) the tumors were isolated according to our tumor isolation protocol (Supplementary Information S3), and FXO_LSFXR_ and FXO_SWFXR_ organoids were established.

### Statistical analysis

Experimental data are given as a mean of (N) independent experiments with (n) replicates. The error bars correspond to the standard deviation (SD). Data analysis was performed using different softwares including: Graphpad Prism v. 8.0.1, Matlab® or RStudio 2022.02.3. Statistical significance (* *p* < 0.05, ** *p* < 0.01 and *** *p* < 0.001) was obtained using, t-test, one- or two-way ANOVA test with multiple comparison tests as indicated in corresponding figure legends.

## Supplementary Information

**Additional file 1.** Supplementary Material.

## Data Availability

All data generated or analysed during this study are included in this published article (and its supplementary information files).

## Abbreviations

3Dcc: 3 Dimensional co-cultures
AUC: Area under the curve
CCI: Computed one-sided confidence interval
CI: Combination index
CMS: Consensus molecular subtype
CRC: Colorectal cancer
CRIS: CRC intrinsic subtype
CUD: Clinically used dose
ECM: Extracellular matrix
FOLFIRI: Chemotherapy combination of folinic acid, 5-Fluorouracil and irinoetcan
FOLFOX: Chemotherapy combination of folinic acid, 5-Fluorouracil and oxaliplatin
FOLFOXIRI: Chemotherapy combination of folinic acid, 5-Fluorouracil, oxaliplatin and irinotecan
mCRC: Metastatic colorectal cancer
MMR: Mismatch repair
MSI: Microsatellite instable
MSS: Microsatellite stable
ODCs: Optimized drug combinations
PDOs: Patient-derived organoids
PTW: Predicted therapeutic window
TGMO: Therapeutically guided multidrug optimization
TME: Tumor micro-environment
TW: Therapeutic window
WES: Whole exome sequencing
