# Supplementary material for: Platform combining statistical modeling and patient-derived organoids to facilitate personalized treatment of colorectal carcinoma
Source: J Exp Clin Cancer Res. 2023 Apr 3;42:79. doi: 10.1186/s13046-023-02650-z (PMC10069117; doi:10.1186/s13046-023-02650-z)
Supplement: Supplementary file 1 — Additional file 1. Supplementary Material. [file 13046_2023_2650_MOESM1_ESM.docx]

# Supplementary Material to :

**Platform combining statistical modeling and patient-derived organoids to facilitate personalized treatment of colorectal carcinoma**

George M. Ramzy^1,2,3^, Maxim Norkin^4^, Thibaud Koessler^5^, Lionel Voirol^6^, Mathieu Tihy^7^, Dina Hany^1,2,3^ Thomas McKee^7^, Frédéric Ris^8^, Nicolas Buchs^8^, Mylène Docquier^9,10^, Christian Toso^11^, Laura Rubbia-Brandt^7^, Gaetan Bakalli^12^, Stéphane Guerrier^2,6^, Joerg Huelsken^4^, Patrycja Nowak-Sliwinska^1,2,3^*

^1^ Molecular Pharmacology Group, School of Pharmaceutical Sciences, University of Geneva, 1211 Geneva, Switzerland

^2^ Institute of Pharmaceutical Sciences of Western Switzerland, University of Geneva, 1211 Geneva, Switzerland

^3^ Translational Research Center in Oncohaematology, 1211 Geneva, Switzerland

^4^ Swiss Institute for Experimental Cancer Research (ISREC), Ecole Polytechnique Fédérale de Lausanne-(EPFL-SV), 1015 Lausanne, Switzerland

^5^ Department of Oncology, Geneva University Hospitals, 1205 Geneva, Switzerland

^6^ Research Center for Statistics, Geneva School of Economics and Management, University of Geneva, 1205 Geneva, Switzerland

^7^ Division of Clinical Pathology, Diagnostic Department, University Hospitals of Geneva (HUG),1205 Geneva, Switzerland

^8^ Translational Department of Digestive and Transplant Surgery, Geneva University Hospitals and Faculty of Medicine, 1205 Geneva, Switzerland

^9^ iGE3 Genomics Platform, University of Geneva, 1211 Geneva, Switzerland

^10^ Department of Genetics & Evolution, University of Geneva, 1211 Geneva, Switzerland

^11^ Department of Visceral Surgery, Geneva University Hospital, 1211 Geneva, Switzerland

^12^ EMLYON Business School, Artificial Intelligence in Management Institute, Ecully, France

**Supplementary Information S1-3**

**Supplementary Tables S1-6**

**Supplementary Figures S1-6**

# Supplementary Information

# Supplementary Information S1: Therapeutically Guided Multidrug Optimization

We employed our proprietary phenotypic approach, the therapeutically guided multidrug optimization (TGMO), to establish drug-drug interactions in initial set of 11 targeted drugs in order to identify synergistic drug combinations (ODC)^1, 2^.

An orthogonal array composite design matrix (OACD) was used to test the different drug combinations. These matrices allow to test a limited number of data points to provide the most optimal information among, among an infinite number of experimental possibilities^3^. OACD matrix is divided into two parts where the first one highlights the linear effect of single and two drugs over a large search space, while the second part exposes the non-linear response of the different drugs over multiple dosage. The screening is performed in three sequential experimental searches, where each search allows the selection of the most active/synergistic drug-drug interactions, while eliminating the most inactive/antagonistic drugs. We initiated our screen by performing drug-dose response curves for all drugs to define their range of activity in both 2D and 3Dcc cultures. This was done in multiple CRC cell lines and non-malignant colon CCD841CoN cells simultaneously, to obtain the therapeutic window, see **Supplementary Figure S1-2**. The latter corresponds to the difference between the activity of both (non-malignant cell viability - cancer cell viability). The output was measured by cell metabolic activity (ATP level, % CTRL). The drug concentrations used are as low as the IC_20_ and half of the latter.

Our mathematical model describes the relationship between the drug combination input and output activity of each possible two-drug interaction, according to:


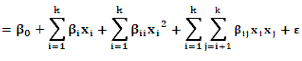


,

Y

where x_i_ and x_j_ are the independent variables (the drugs) and ε is a random error term. The activity is equal to the sum of β_0_ (the intercept), β_i,_(linear term) β_ii_ (quadratic term) and β_ij_ (interaction term)^4^.

The regression coefficients describe (i) the contribution of each drug individually (single drug first-order term) (ii) all drug-drug interactions, and (iii) how the effect of a drug varies over different dose levels (single drug second-order terms). The generated models guide drug selection and elimination, as the negative regression coefficients in (i) and (ii) are considered as synergistic activity, and positive regression coefficients signify antagonistic activity. Whereas for (iii), a positive value of regression coefficient corresponds to a stable effect over the dose range tested, and negative regression coefficient highlights a dose-dependent contributions. Through consecutive rounds of screening (search 1-3) the most robust drug interactions define the final drug combination selection.

**Supplementary Information S2: Adaptive lasso**

In this study, cross-validated our findings obtained from the TGMO-based screen with another mathematical predictive model called adaptive lasso.

The same experimental data as defined by TGMO-based matrices, were delivered to initiates adaptive lasso^5^. The main objective was to find the combination of $q$ drugs (as well as their respective dosage) corresponding to the highest therapeutic windows. For simplicity, we let $\mathbf{Y}_{1}$ and $\mathbf{Y}_{2}$ denote, respectively, the vectors of measured cell viability of cancer and healthy cells obtained in various experimental conditions. To evaluate the therapeutic window of all possible drug combinations, we consider the following linear regression setting:

$$\mathbf{Y}_{i}=\alpha_{i}+\mathbf{X}_{i}\boldsymbol{\beta}_{i}+\boldsymbol{\varepsilon}_{i},$$

for $i=1,2$. The vectors of coefficients $\boldsymbol{\beta}_{i}$ are, respectively, associated to cell viability of cancer cells and healthy cells. The intercepts $\alpha_{i}$ corresponds to expected cell viability (of cancer cells and healthy cells, respectively) in the absence of drugs. Moreover, we assumed that $\varepsilon_{i}$ is a mean-zero random vector composed of independent and identically distributed element with variance $\sigma_{i}^{2}$. The distribution of the elements $\varepsilon_{i}$ was left unspecified. The matrices $\mathbf{X}_{i}$ encode the different experimental conditions such that its elements, which combination of drugs and dosages were tested. These matrices incorporate the marginal effects of each drug/dosage as well as all $q$-(th) order interactions between drugs in the matrix $\mathbf{X}_{i}$. The interactions between the same drug at different dosages were excluded. More specifically, the $\left( k,j \right)$-th element of $\mathbf{X}_{i}$ is equal to $1$ if the $k$-th experiment (corresponding to the $k$-th element of $\mathbf{Y}_{i}$) included the drug/dosage or combination of drugs/dosages associated with the $j$-th column of $\mathbf{X}_{i}$. In our setting, we consider the interactions up to $q=4$ drugs/dosages and we have $\mathbf{X}_{1}=\mathbf{X}_{2}$.

Considering the above-setting, the dimension of the $\boldsymbol{\beta}_{i}$, say $p$, grows exponentially with $q$ (i.e., the number of considered drug combinations) and in practice this quantity considerably exceeds the number of experiments $n$ (i.e., the dimensions of $\mathbf{Y}_{i}$). Consequently, the standard ordinary least squares estimator cannot be computed in this case as $p>n$. To circumvent this limitation, we considered a penalized estimator, which can provide reliable estimates in situations where $p>n$ but under the implicit assumption that the true parameter vector $\boldsymbol{\beta}_{i}$ (i.e., an important proportion of the elements of $\boldsymbol{\beta}_{i}$ are equal to zero). More precisely, we considered a data-depended adaptive version of the lasso proposed by Hui Zou in 2006^5^. This regression method is based on an initial estimator (i.e., the standard lasso of Tibshirani (1996)^6^ using cross-validation), which is used through data driven weights in the adaptive Lasso procedure. More specifically, we computed the weights in a such way that they consider some features of the data, i.e. the marginal effect has more importance than the first-order interactions, which are more significant that the second-order interactions, etc.

To evaluate the therapeutic windows of all possible combinations of $q$ drugs (at specific dosages), we constructed $m$ vectors of dimension $p$ such the $j$-th vector (where $1\leq j\leq m)$, where $\mathbf{w}_{j}$ encodes the drugs/dosages of the $j$-th combination. Thus, $m$ denoted the total number of possible combinations. Denoting as ${\hat{\boldsymbol{\beta}}}_{i}$ as the estimated parameter vectors of the adaptive lasso procedure, we expressed the predicted therapeutic window (PTW) of the $j$-th combination as follows:

$$\text{PTW}_{j}={\hat{\mathbf{Y}}}_{2}-{\hat{\mathbf{Y}}}_{1}=\mathbf{w}_{j}\left( {\hat{\boldsymbol{\beta}}}_{2}-{\hat{\boldsymbol{\beta}}}_{1} \right),$$

where ${\hat{\mathbf{Y}}}_{1}$ and ${\hat{\mathbf{Y}}}_{2}$ denoted, respectively, the predicted cell viability of cancer cell and healthy cell. Moreover, under suitable conditions, as shown by Sen *et al.^7^*, it can be noted that an approximate $1-\alpha$ confidence intervals (with $0<\alpha<1$) can be computed as follows:

$$\text{PTW}_{j}\pm z_{1-\alpha/2}\hat{\sigma}_{j},$$

where $z_{1-\alpha/2}$ denoted the $1-\alpha/2$ quantile of a standard normal distribution, while $\hat{\sigma}_{j}$ corresponded to estimated standard deviation of $\text{PTW}_{j}$. Unfortunately, these confidence intervals are rough estimates of the uncertainty associated $\text{PTW}_{j}$ as they ignore the issue of known as inference after selection and relies on asymptotic theory. However, this simple approach can be justified to some extent from theoretical standpoint^7^. While the problem of inference after selection is still an open question in statistics, we considered this procedure as optimistic, since it might reject a treatment that might be equivalent to the one that maximizes the PTW, hence being conservative.

To identify the best combination of $q$ drugs (as well as their respective dosage) corresponding to the highest therapeutic windows, we considered the order statistic of $\text{PTW}_{\left( j \right)}$ so that for all $1\leq j<k\leq m$ we have $\text{PTW}_{\left( j \right)}\leq\text{PTW}_{\left( k \right)}$. A heuristic approach would construct an asymmetric confidence interval around the maximum value of the PTW, and select the set of drug/dosage combinations that cannot be differentiated from the treatment that maximizes the PTW. Hence, the result is a set of drug combinations with indistinguishable optimal performance.

# Supplementary Information S3: Tumor isolation protocol

Freshly isolated tissue samples were placed in 20 mL of transport media (DMEM/F12 + primocin) immediately after tumor resection. After delivery to the laboratory, the storage medium was discarded, and tissues were washed with 20 mL HBSS by active inverting of the tube. After the HBSS wash solution was discarded, the sample was transferred to a new culture dish. The tissue was then minced with forceps or razor blades into small (1-2 mm) pieces, in 1ml of digestion medium (DMEM/F12 + Liberase DH (0.28 units/ml)). Tissue pieces were transferred to GentleMACS violet C tubes. 10 mL/tube/gr of tissue (minimal 5 mL/tube) were used and the protocol 37C_TDK-01 was used for 1h. In the next step, the samples were filtered with a 40 μm cell strainer.

The retained fragments of tissue remaining in the cell strainer were collected using a pipetman and resuspend in 10 mL HBSS. Then, centrifugation at 1200 RPMI at 4°C for 5 min was performed. The samples were then washed with 20 mL HBSS by inverting the tube and centrifuged at 1200 RPMI at 4°C for 5 min. The washing solution was then discarded, and the material was then resuspended in the adequate volume of Matrigel^®^ in a serum-free stem cell medium (DMEM/F12 + StemPro hESC) supplemented with 8 ng/mL bFG in 6-WP. Incubation in a 5% CO_2_-humidified incubator at 37°C was performed for 20 min, the time for the Matrigel^®^ to polymerize, then 3 ml of culture medium were added on top.

# Supplementary Information S4: Immunohistochemistry

Single organoids were pooled from 96-WP, for both CTRL and ODC_LSFXR_ treated conditions, and fixed in cold PFA (4%) for 3 hours, then washed and kept in PBS at 4°C. A 2% agarose solution was prepared, and the single organoids were embedded in a drop of the solution, by forming a solidified dome on top.

Finally, the agar domes were then embedded in paraffin and the different blocks were sectioned every 5µm using a microtome. The obtained slices were mounte on glass slides and put in a humidifier chamber until staining. The different slides were then stained according to the routinely performed hematoxylin and eosin (H&E) staining protocol. The slides were analyzed with the widefield Axioscan Z1, Zeiss® microscope and ZEN® software.

# Supplementary Tables

# Supplementary Table S1. CRC cell lines used in the study and their characterization^8-12^

| **Cell line** | **Patient** | **Cancer Stage/Type** | **Genomic (In)stability** | **Consensus Molecular Subtype** | **Mutations/Deregulations** |
| --- | --- | --- | --- | --- | --- |
| **LS174T** | Female | 2 (primary) | *MSI* | *CMS3* | *KRAS, PIK3CA, BRAF* |
| **SW620** | Male | 3 (metastatic) | *MSS; CIN* | *CMS4* | *APC, KRAS, TP53* |

**Supplementary Table S2. Combination index (CI) of all drug-drugs interactions of the final ODC**

| **SW620** | **LS174T** | **SWFXR** | **LSFXR** |
| --- | --- | --- | --- |
| Reg+Vem (0.53)  Reg+Vat (0.32)  Reg+AZD (0.40)  Vem+Vat (0.47)  Vem+AZD (0.55)  Vat+AZD (0.23) | Erl+Lap (0.23)  Erl+Palb (0.10)  Erl+Nilo (1.1)  Lap+Palb (0.10)  Lap+Nilo (0.40)  Palb+Nilo (0.34) | Reg+Lap (0.11)  Reg+Palb (0.03)  Reg+AZD (1.1)  Lap+Palb (0.008)  Lap+AZD (0.007)  Palb+AZD (0.01) | Reg+Vem (0.78)  Reg+Palb (0.32)  Reg+Lap (0.97)  Vem+Palb (0.38)  Vem+Lap (0.48)  Palb+Lap (0.14) |

Drugs: Reg, regorafenib, Vem, vemurafenib; Vat, vatalanib; AZD, AZD-4547; Erl, erlotinib; Lap, lapatinib; Palb, palbociclib; Nilo, nilotinib. CI < 1 = synergistic combination, CI > 1 = antagonistic combination.

**Supplementary Table S3. Computed confidence interval (CCI) in CRC 3Dcc_SW620_ models**

| Drug Combinations in CCI | Pred. Healthy (%) | Pred. Cancer (%) | PTW (%) |
| --- | --- | --- | --- |
| azd45472-nilotinib2-regorafenib2-vemurafenib2 | 45.02 | 81.05 | 36.03 |
| azd45472-nilotinib1-regorafenib2-vemurafenib2 | 43.14 | 82.55 | 39.41 |
| azd45472-regorafenib2-vatalanib2-vemurafenib2 | 41.98 | 75.35 | 33.37 |

**Supplementary Table S4. Computed confidence interval (CCI) in CRC 3Dcc_LS174T_ models**

| Drug Combinations in CCI | Pred. Healthy (%) | | Pred. Cancer (%) | PTW (%) | |
| --- | --- | --- | --- | --- | --- |
| erlotinib2-lapatinib1-nilotinib1-palbociclib1 | 91.29 | 63.30 | | | 27.98 |
| erlotinib1-lapatinib1-nilotinib1-palbociclib1 | 92.30 | 65.29 | | | 27.01 |
| lapatinib1-nilotinib1-palbociclib1-trametinib2 | 92.30 | 65.93 | | | 26.37 |
| lapatinib1-nilotinib1-palbociclib1-trametinib1 | 96.11 | 69.85 | | | 26.26 |
| erlotinib2-lapatinib2-nilotinib1-palbociclib1 | 91.29 | 65.74 | | | 25.54 |
| Lapatinib1 -nilotinib1-palbociclib1-vatalanib1 | 92.30 | 66.97 | | | 25.32 |
| erlotinib2-lapatinib1-nilotinib2-palbociclib1 | 90.72 | 65.46 | | | 25.26 |
| lapatinib1-nilotinib1-palbociclib1-vatalanib2 | 91.11 | 66.04 | | | 25.07 |
| lapatinib1-nilotinib1-olaparib2-palbociclib1 | 90.93 | 65.96 | | | 24.97 |
| erlotinib1-lapatinib2-nilotinib1-palbociclib1 | 92.30 | 67.73 | | | 24.57 |
| erlotinib1-lapatinib1-nilotinib2-palbociclib1 | 91.73 | 67.45 | | | 24.28 |
| lapatinib2-nilotinib1-palbociclib1-trametinib2 | 92.30 | 68.37 | | | 23.93 |
| lapatinib2-nilotinib1-palbociclib1-trametinib1 | 96.11 | 72.29 | | | 23.82 |
| lapatinib1-nilotinib2-palbociclib1-trametinib2 | 91.73 | 68.09 | | | 23.64 |
| lapatinib1-nilotinib2-palbociclib1-trametinib1 | 95.54 | 72.01 | | | 23.53 |
| lapatinib2-nilotinib1-palbociclib1-vatalanib1 | 92.30 | 69.41 | | | 22.89 |
| bez2351-lapatinib1-nilotinib1-palbociclib1 | 85.42 | 62.58 | | | 22.84 |
| erlotinib2-lapatinib2-nilotinib2-palbociclib1 | 90.72 | 67.90 | | | 22.82 |
| lapatinib2-nilotinib1-palbociclib1-vatalanib2 | 91.11 | 68.48 | | | 22.63 |
| lapatinib1-nilotinib2-palbociclib1-vatalanib1 | 91.73 | 69.13 | | | 22.60 |
| erlotinib2-lapatinib1-nilotinib1-palbociclib2 | 84.61 | 62.06 | | | 22.56 |
| lapatinib2-nilotinib1-olaparib2-palbociclib1 | 90.93 | 68.40 | | | 22.53 |
| lapatinib1-nilotinib2-palbociclib1-vatalanib2 | 90.55 | 68.20 | | | 22.35 |
| lapatinib1-nilotinib2-olaparib2-palbociclib1 | 90.36 | 68.12 | | | 22.25 |
| erlotinib1-lapatinib2-nilotinib2-palbociclib1 | 91.73 | 69.89 | | | 21.84 |
| erlotinib1-lapatinib1-nilotinib1-palbociclib2 | 85.63 | 64.04 | | | 21.58 |
| lapatinib2-nilotinib2-palbociclib1-trametinib2 | 91.73 | 70.53 | | | 21.20 |
| lapatinib2-nilotinib2-palbociclib1-trametinib1 | 95.54 | 74.45 | | | 21.09 |
| azd45472-lapatinib1-nilotinib1-palbociclib1 | 86.42 | 65.39 | | | 21.03 |
| lapatinib1-nilotinib1-palbociclib2-trametinib2 | 85.63 | 64.69 | | | 20.94 |
| lapatinib1-nilotinib1-palbociclib2-trametinib1 | 89.44 | 68.61 | | | 20.83 |
| erlotinib2-nilotinib1-palbociclib1-trametinib2 | 91.29 | 70.61 | | | 20.68 |
| lapatinib1-nilotinib1-olaparib1-palbociclib1 | 88.76 | 68.08 | | | 20.67 |
| erlotinib2-nilotinib1-palbociclib1-trametinib1 | 95.10 | 74.53 | | | 20.57 |
| bez2351-lapatinib2-nilotinib1-palbociclib1 | 85.42 | 65.02 | | | 20.40 |
| lapatinib1-nilotinib1-palbociclib2-regorafenib2 | 76.01 | 55.72 | | | 20.29 |
| lapatinib2-nilotinib2-palbociclib1-vatalanib1 | 91.73 | 71.57 | | | 20.16 |
| erlotinib2-lapatinib2-nilotinib1-palbociclib2 | 84.61 | 64.50 | | | 20.12 |
| bez2351-lapatinib1-nilotinib2-palbociclib1 | 84.85 | 64.74 | | | 20.12 |
| lapatinib2-nilotinib2-palbociclib1-vatalanib2 | 90.55 | 70.64 | | | 19.91 |
| lapatinib1-nilotinib1-palbociclib2-vatalanib1 | 85.63 | 65.73 | | | 19.90 |
| erlotinib2-lapatinib1-nilotinib2-palbociclib2 | 84.05 | 64.22 | | | 19.83 |
| lapatinib2-nilotinib2-olaparib2-palbociclib1 | 90.36 | 70.56 | | | 19.81 |
| erlotinib1-nilotinib1-palbociclib1-trametinib2 | 92.30 | 72.59 | | | 19.71 |
| lapatinib1-nilotinib1-palbociclib2-vatalanib2 | 84.44 | 64.80 | | | 19.65 |
| erlotinib2-nilotinib1-palbociclib1-vatalanib1 | 91.29 | 71.65 | | | 19.64 |
| azd45471-lapatinib1-nilotinib1-palbociclib1 | 88.80 | 69.18 | | | 19.62 |
| erlotinib1-nilotinib1-palbociclib1-trametinib1 | 96.11 | 76.51 | | | 19.60 |
| lapatinib1-nilotinib1-olaparib2-palbociclib2 | 84.26 | 64.71 | | | 19.55 |
| erlotinib2-nilotinib1-palbociclib1-vatalanib2 | 90.10 | 70.72 | | | 19.39 |
| erlotinib2-nilotinib1-olaparib2-palbociclib1 | 89.92 | 70.63 | | | 19.29 |
| erlotinib1-lapatinib2-nilotinib1-palbociclib2 | 85.63 | 66.48 | | | 19.14 |
| lapatinib1-nilotinib1-palbociclib1-regorafenib2 | 82.68 | 63.55 | | | 19.13 |
| erlotinib2-lapatinib1-palbociclib1-trametinib2 | 83.51 | 64.42 | | | 19.09 |
| bez2352-lapatinib1-nilotinib1-palbociclib1 | 77.41 | 58.34 | | | 19.07 |
| erlotinib2-lapatinib1-palbociclib1-trametinib1 | 87.32 | 68.34 | | | 18.98 |
| erlotinib1-lapatinib1-nilotinib2-palbociclib2 | 85.06 | 66.20 | | | 18.86 |

**Supplementary Table S5: Computed confidence interval (CCI) in CRC 3D-FX_SW620_ model**

| Drug Combinations in CCI | Pred. Cancer (%) |
| --- | --- |
| palbociclib1-regorafenib2-trametinib2-vemurafenib2 | 16.76 |
| lapatinib1-palbociclib2-regorafenib2-vemurafenib2 | 18.35 |
| lapatinib2-palbociclib1-regorafenib2-vemurafenib2 | 19.95 |
| lapatinib2-palbociclib2-regorafenib2-vemurafenib1 | 21.82 |

**Supplementary Table S6. Computed confidence interval (CCI) in CRC 3D-FX_LS174T_ model**

| Drug Combinations in CCI | Pred. Cancer (%) |
| --- | --- |
| lapatinib2-palbociclib2-regorafenib2-vemurafenib2 | 30.95 |
| azd45472-lapatinib2-palbociclib2-regorafenib2 | 32.50 |
| lapatinib2-palbociclib2-regorafenib2-vemurafenib1 | 35.71 |
| azd45471-lapatinib2-palbociclib2-regorafenib2 | 36.19 |
| lapatinib2-palbociclib2-regorafenib2-trametinib2 | 38.59 |

# Supplementary Figures

**
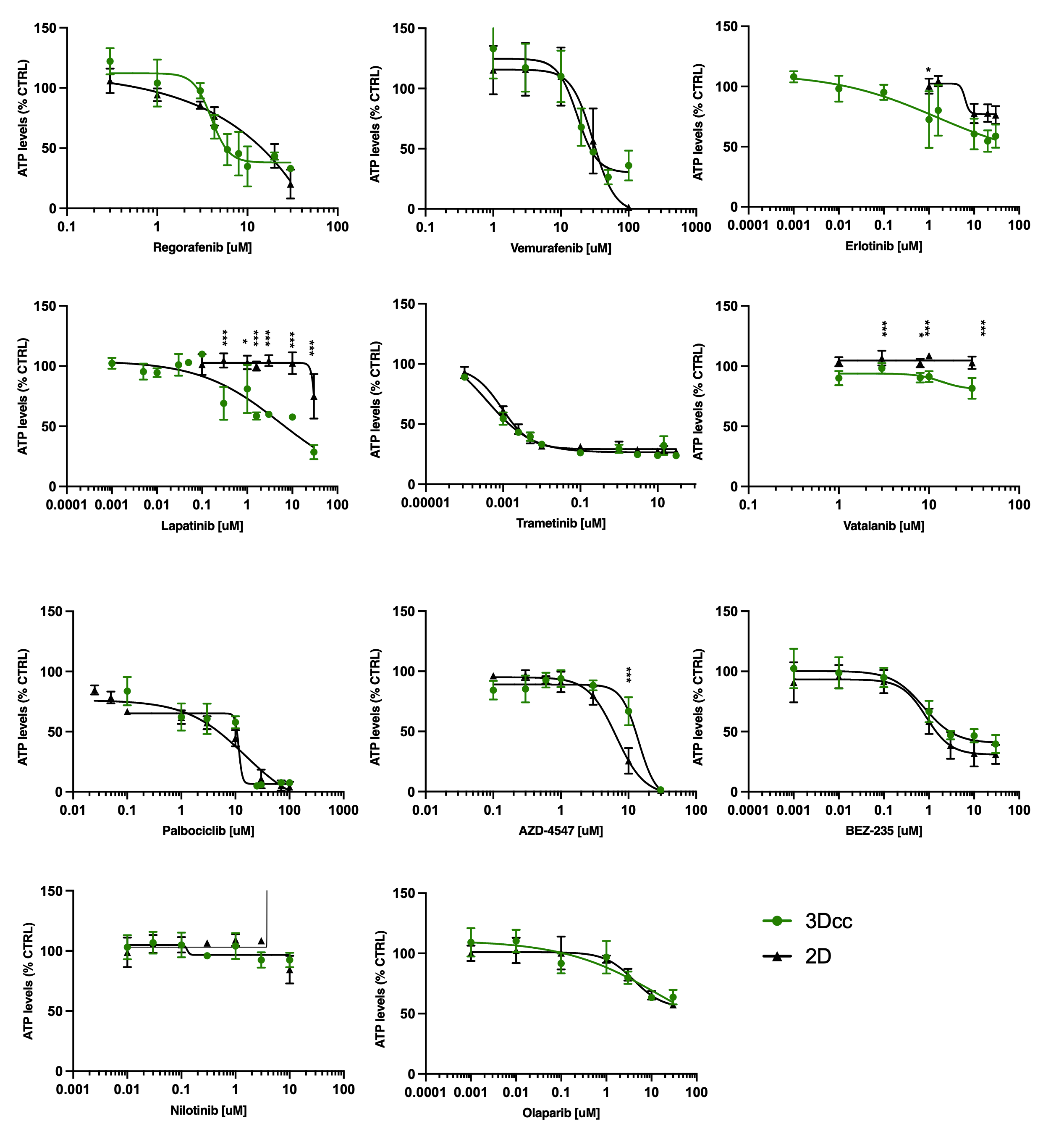
Supplementary Figure S1. Drug-dose response curves in SW620-based cell models.**

Drug-dose response curves of all eleven drugs used in the TGMO based screen, performed on SW620 2D cultures (black curve) vs. 3Dcc_SW620_ (green curves). Drug concentrations are presented on a logarithmic scale. Data is presented as the mean of N=3 independent experiments, and error bars represent the SD. Significance is determined by two-way ANOVA with *p<0.05, **p<0.01 and ***p<0.001 representing the comparison between the drug-response in 2D_SW620_ vs. 3Dcc_SW620_.

**
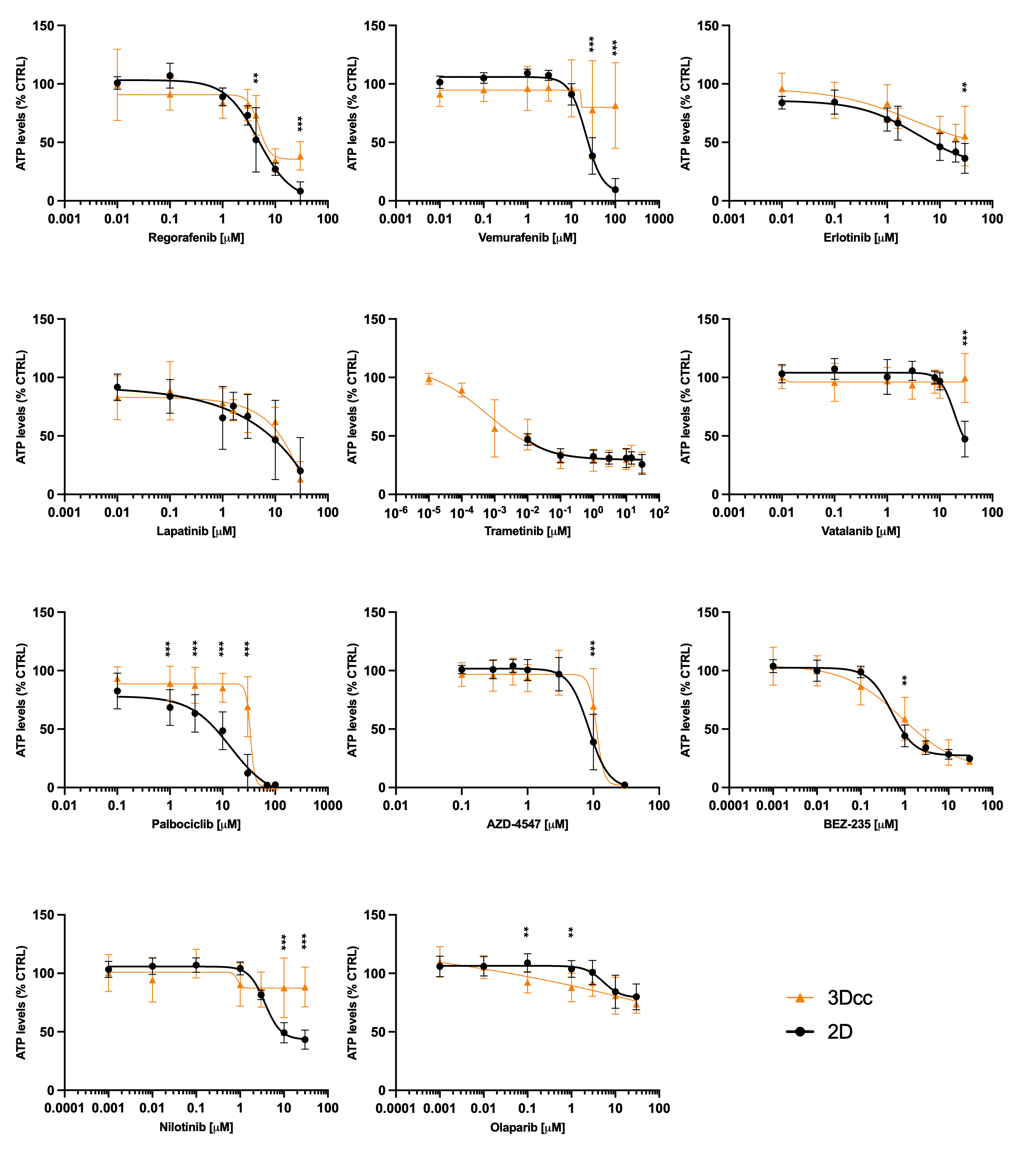
Supplementary Figure S2. Drug-dose response curves in LS174T-based cell models**

Drug-dose response curves of all eleven drugs used in the TGMO based screen, performed on SW620 2D cultures (black curve) vs. 3Dcc_LS174T_ (orange curves). Drug concentrations are presented on a logarithmic scale. Data is presented as the mean of N=3 independent experiments, and error bars represent the SD. Significance is determined by two-way ANOVA with *p<0.05, **p<0.01 and ***p<0.001 representing the comparison between the drug-response in 2D_LS174T_ vs. 3Dcc_LS174T_.

#

# Supplementary Figure S3. TGMO-based screen in 3Dcc_SW620_

Regression coefficients of single drug 1^st^ order, 2-drugs, and single drug 2^nd^ order drug interactions (red, burgundy, and pink lines respectively) in *Search 1* **(A)**, screening of 155 drug combinations with 11 drugs (n=3, N=3). **B.** *Search 2*, screening of 50 drug combinations with 7 drugs (n=3, N=3).

**C.** *Search 3*, screening of 25 drug combinations with 4 drugs (n=3, N=2). The screening was performed in 3Dcc_SW620_ (green striped bars) and the therapeutic window TW (black bars) generated from 3Dcc_CCD841_. Accuracy of the model is given by the R^2^. Error bars represent the SD and significance of estimated regression coefficients was determined with a one-way ANOVA and is represented with *p < 0.05, **p < 0.01 and 0.01 < ***p < 0.001.

#

# Supplementary Figure S4. TGMO-based screen in 3Dcc_LS174T_

Regression coefficients of single drug 1^st^ order, 2-drugs, and single drug 2^nd^ order drug interactions (red, burgundy, and pink lines respectively) in **A.** *Search 1*, screening of 155 drug combinations with 11 drugs (n=3, N=3). **B.** *Search 2*, screening of 50 drug combinations with 7 drugs (n=3, N=2). **C.** *Search 3*, screening of 25 drug combinations with 4 drugs (n=3, N=2). The screening was performed in 3Dcc_LS174T_ (orange striped bars) and the therapeutic window TW (black bars) generated from 3Dcc_CCD841_. Accuracy of the model is given by the R^2^. Error bars represent the SD and significance of estimated regression coefficients was determined with a one-way ANOVA and is represented with *p < 0.05, **p < 0.01 and 0.01 < ***p < 0.001.

#

# Supplementary Figure S5. TGMO-based screen in FXO_SWFXR_ and FXO_LSFXR_

Regression coefficients of single drug 1^st^ order, 2-drugs, and single drug 2^nd^ order drug interactions (red, burgundy, and pink lines respectively) in **A-C.** *Search 1*, screening of 50 drug combinations with 7 drugs (n=3, N=3). **B-D.** *Search 2*, screening of 25 drug combinations with 4 drugs (n=3, N=2). The screening was performed in FXO_SW620_ (green squared bars) and FXO_LS174T_ (orange squared bars) respectively. Accuracy of the model is given by the R^2^. Error bars represent the SD and significance of estimated regression coefficients was determined with a one-way ANOVA and is represented with *p < 0.05, **p < 0.01 and 0.01 < ***p < 0.001.

**
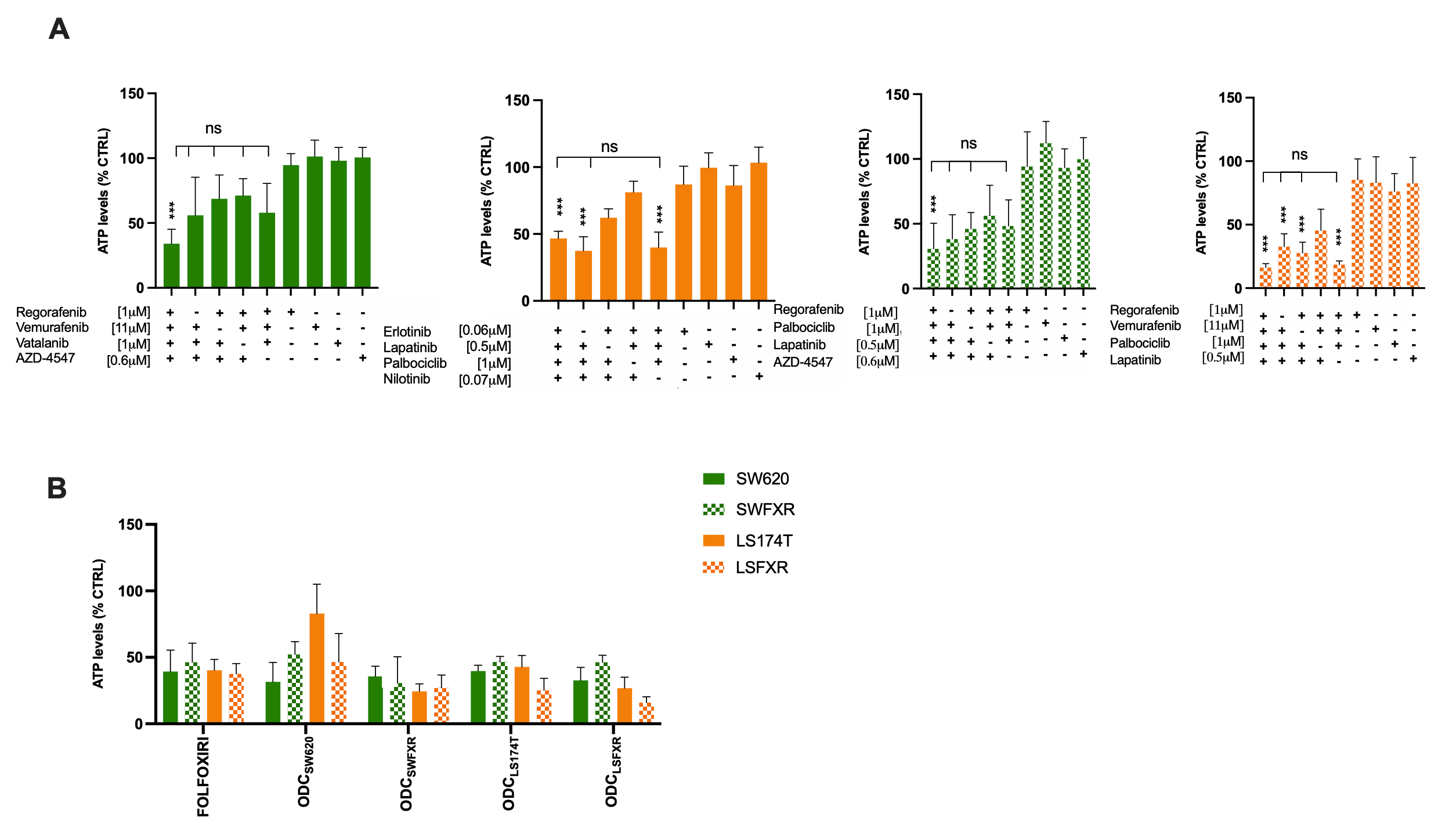
**

# Supplementary Figure S6 - Cross-validation of ODC activity in CRC 3D models

**A.** Activity of ODCs, vs. activity obtained by removing a drug from the ODCs vs all corresponding monotherapies in 3Dcc_SW620_ (in green), 3DccLS174T (in orange), 3D-FX_SWFXR_ (squared green) and 3D-FX_LSFXR_ (squared orange) (n=3, N=3). **B.** Cross-validation of the cell-specific ODCs across the panel of CRC 3D models used vs. and FOLFOXIRI (folinic acid [0.5µM], 5-FU [10µM], SN38 [0.1µM] and oxaliplatin [0.5µM]). Activity is measured by ATP levels vs. CTRL (<0.15% DMSO). Error bars represent SD. Significance is determined by two-way ANOVA with *p<0.05, **p<0.01 and ***p<0.001.

# Supplementary Figure S7 – H&E staining

# Representative images of H&E staining of tissue sections from the primary tumor tissue (left panels), and corresponding single organoids in CTRL (middle panels) and ODC_LSFXR_-treated conditions (right panels) for A. PCRC-2 and B. PCRC-3. Morphological similarities are observed between the patient tumor tissue and corresponding single organoid. Scale bar = 100 μm.

**Supplementary references**

1. Weiss, A.; Le Roux-Bourdieu, M.; Zoetemelk, M.; Ramzy, G. M.; Rausch, M.; Harry, D.; Miljkovic-Licina, M.; Falamaki, K.; Wehrle-Haller, B.; Meraldi, P., et al., Identification of a Synergistic Multi-Drug Combination Active in Cancer Cells via the Prevention of Spindle Pole Clustering. *Cancers (Basel)* **2019,** *11* (10).

2. Zoetemelk, M.; Ramzy, G. M.; Rausch, M.; Koessler, T.; van Beijnum, J. R.; Weiss, A.; Mieville, V.; Piersma, S. R.; de Haas, R. R.; Delucinge-Vivier, C., et al., Optimized low-dose combinatorial drug treatment boosts selectivity and efficacy of colorectal carcinoma treatment. *Mol Oncol* **2020,** *14* (11), 2894-2919.

3. Xu, H.; Jaynes, J.; Ding, X., COMBINING TWO-LEVEL AND THREE-LEVEL ORTHOGONAL ARRAYS FOR FACTOR SCREENING AND RESPONSE SURFACE EXPLORATION. *Statistica Sinica* **2014,** *24* (1), 269-289.

4. Weiss, A.; Berndsen, R. H.; Ding, X.; Ho, C. M.; Dyson, P. J.; van den Bergh, H.; Griffioen, A. W.; Nowak-Sliwinska, P., A streamlined search technology for identification of synergistic drug combinations. *Sci Rep* **2015,** *5*, 14508.

5. Zou, H., The Adaptive Lasso and Its Oracle Properties. *Journal of the American Statistical Association* **2006,** *101* (476), 1418-1429.

6. Tibshirani, R., Regression Shrinkage and Selection via the Lasso. *Journal of the Royal Statistical Society. Series B (Methodological)* **1996,** *58* (1), 267-288.

7. Sen, Z.; Daniela, W.; Ali, S., In Defense of the Indefensible: A Very Naïve Approach to High-Dimensional Inference. *Statistical Science* **2021,** *36* (4), 562-577.

8. Ahmed, D.; Eide, P. W.; Eilertsen, I. A.; Danielsen, S. A.; Eknaes, M.; Hektoen, M.; Lind, G. E.; Lothe, R. A., Epigenetic and genetic features of 24 colon cancer cell lines. *Oncogenesis* **2013,** *2*, e71.

9. Chen, T. R.; Dorotinsky, C. S.; McGuire, L. J.; Macy, M. L.; Hay, R. J., DLD-1 and HCT-15 cell lines derived separately from colorectal carcinomas have totally different chromosome changes but the same genetic origin. *Cancer genetics and cytogenetics* **1995,** *81* (2), 103-8.

10. Berg, K. C. G.; Eide, P. W.; Eilertsen, I. A.; Johannessen, B.; Bruun, J.; Danielsen, S. A.; Bjørnslett, M.; Meza-Zepeda, L. A.; Eknæs, M.; Lind, G. E., et al., Multi-omics of 34 colorectal cancer cell lines - a resource for biomedical studies. *Mol Cancer* **2017,** *16* (1), 116-116.

11. Tom, B. H.; Rutzky, L. P.; Jakstys, M. M.; Oyasu, R.; Kaye, C. I.; Kahan, B. D., Human colonic adenocarcinoma cells. I. Establishment and description of a new line. *In vitro* **1976,** *12* (3), 180-91.

12. Brattain, M. G.; Fine, W. D.; Khaled, F. M.; Thompson, J.; Brattain, D. E., Heterogeneity of malignant cells from a human colonic carcinoma. *Cancer Res* **1981,** *41* (5), 1751-6.
